# Methylation-Based Signatures for Gastroesophageal Tumor Classification

**DOI:** 10.3390/cancers12051208

**Published:** 2020-05-11

**Authors:** Nikolay Alabi, Dropen Sheka, Ashar Siddiqui, Edwin Wang

**Affiliations:** 1 Department of Biochemistry and Molecular Biology, Faculty of Medicine, University of Calgary, Calgary, Alberta T2N 1N4, Canada; nikolay.alabi@ucalgary.ca; 2 Cumming School of Medicine, University of Calgary, Calgary, Alberta T2N 1N4, Canada; Ashar.siddiqui1@ucalgary.ca

**Keywords:** Multi-Survival Screening Algorithm, MSS, methylation array-based profile, gastroesophageal junction cancer, predictor, gastric cancer, esophageal cancer, methylation signature, tumor classification, gastroesophageal cancer diagnosis, tumor characterization

## Abstract

Contention exists within the field of oncology with regards to gastroesophageal junction (GEJ) tumors, as in the past, they have been classified as gastric cancer, esophageal cancer, or a combination of both. Misclassifications of GEJ tumors ultimately influence treatment options, which may be rendered ineffective if treating for the wrong cancer attributes. It has been suggested that misclassification rates were as high as 45%, which is greater than reported for junctional cancer occurrences. Here, we aimed to use the methylation profiles of GEJ tumors to improve classifications of GEJ tumors. Four cohorts of DNA methylation profiles, containing ~27,000 (27k) methylation sites per sample, were collected from the Gene Expression Omnibus and The Cancer Genome Atlas. Tumor samples were assigned into discovery (n_EC_ = 185, n_GC_ = 395; EC, esophageal cancer; GC gastric cancer) and validation (n_EC_ = 179, n_GC_ = 369) sets. The optimized Multi-Survival Screening (MSS) algorithm was used to identify methylation biomarkers capable of distinguishing GEJ tumors. Three methylation signatures were identified: They were associated with protein binding, gene expression, and cellular component organization cellular processes, and achieved precision and recall rates of 94.7% and 99.2%, 97.6% and 96.8%, and 96.8% and 97.6%, respectively, in the validation dataset. Interestingly, the methylation sites of the signatures were very close (i.e., 170–270 base pairs) to their downstream transcription start sites (TSSs), suggesting that the methylations near TSSs play much more important roles in tumorigenesis. Here we presented the first set of methylation signatures with a higher predictive power for characterizing gastroesophageal tumors. Thus, they could improve the diagnosis and treatment of gastroesophageal tumors.

## 1. Introduction

As each cancer type contains different characteristics and, therefore, requires a unique approach for treatment, the clinical management process of cancer in patients is highly dependent on how a cancer is identified. Today, contention exists among both physicians and researchers as to whether gastroesophageal cancer is the same as gastric or esophageal cancer, if it is a combination of the two, or if gastroesophageal cancer is a new type altogether [1]. Although gastric, esophageal, and gastroesophageal junction (GEJ) tumors share some common biological behaviors, they display distinct risk factors, molecular mechanisms, and histological types [2,3,4,5]. Therefore, it is necessary to have a better understanding of the unique molecular differences of these tumors as a critical step towards understanding the biology and improving our currently limited management approaches [6,7].

In the past, anatomical classification systems such as the Siewert’s classification system have been applied to classify gastroesophageal tumors [2]. Today, the eighth edition of the Union for International Cancer Control-American Joint Committee on Cancer (UICC-AJCC) classification system associates gastroesophageal tumors and esophageal tumors within the same group [1,4]. This staging system directly favors guidelines for the best course of treatment options to revolve around esophageal tumor management, as opposed to gastric tumor management approaches for patients experiencing gastroesophageal cancer variants. The concern with treating gastroesophageal tumors with esophageal tumor treatments is that this may not be the best course of action if the tumor has more gastric tumor attributes as compared to esophageal. A strong example of this is present in the differences of approach to chemoradiotherapy treatment of gastric tumors versus esophageal tumors. Only a handful of studies demonstrate a significant survival advantage for neoadjuvant chemotherapy; in general, post-operative chemotherapy is not recommended for esophageal tumors [8,9,10]. Contrastingly, in gastric tumor treatment there has been a strong push towards the utilization of effective chemotherapy and radiation regimens to be used with surgical management and various trials have demonstrated a significant improvement in resectability, progression-free survival, and overall survival in patients with gastric and GEJ tumors given perioperative chemotherapy compared to surgery alone [11,12]. Thus, treating gastroesophageal tumors simply as esophageal tumors may have negative consequences on the effectiveness of the treatment plan the patient receives if the tumor has gastric attributes or if a tumor diagnosed as gastric has esophageal attributes. Furthermore, the misclassification of tumors has consequently resulted in a fraction of esophageal and gastric adenocarcinomas being diagnosed as GEJ tumors and vice versa [13]. Although it is difficult to predict the extent to which misclassification has affected incidence rates for GEJ carcinoma, a Swedish study estimated that low accuracy in registering junctional cancers has resulted in true incidence rates being up to 45% greater than reported [13,14]. While an esophageal adenocarcinoma diagnosed as a GEJ tumor would typically not have any detrimental implications since the general treatments are similar, a gastric adenocarcinoma being diagnosed as a GEJ tumor and an esophageal tumor being diagnosed as a gastric tumor can have a consequential impact on the effectiveness of the treatment as therapies for the two vary, with treatments for GEJ tumors centering around esophageal tumor treatments. The diagnosis of the type of cancer the patient is diagnosed with has an impact on the prognosis, which subsequently impacts the treatment plan. Thus, there is a need for an improved method for the characterization and classification of gastroesophageal tumors found in the gastroesophageal tract in order to provide more accurate and effective diagnoses to inform prognostic and treatment options.

Molecular characteristics in the form of messenger RNA (mRNA) and microRNA (miRNA) expression profiles have emerged as valuable biomarkers/tools for tumor signature characterizations of various cancer types [1,15,16,17]. Conventionally, gene expression signatures to determine the origin of the cells have been used for tumor classification [1,18]. However, Spainhour et al. [18] found that, at the gene level of many cancer types, different methylation patterns can yield similar gene expressions. Therefore, patterns can be better visualized by analyzing methylation as opposed to gene expression. While gene expression and miRNA signatures that can distinguish gastric adenocarcinomas from esophageal carcinomas exist [1,19], methylation profiles are of merit to investigate in order to consolidate molecular characterizations of gastroesophageal tumors. Robust genetic molecular biomarker(s) of this variant are yet to be established to serve this purpose. While there are tumor methylation markers tailored to esophageal and gastric cancer separately, there lacks a robust methylation tissue marker that can distinguish between the cancer types in GEJ tumors [20,21,22]. Thus, it is necessary to identify a universal methylation signature with the goal of pursuing more individualized treatments options through better classification of gastroesophageal tumors.

## 2. Methods

### 2.1. Data Processing and Normalization

The following four methylation array-based gene expression profiles were collected from the repositories of Gene Expression Omnibus (GEO) and The Cancer Genome Atlas (TCGA): (1) TCGA Esophageal Cancer (ESCA), comprised of 185 samples using the Illumina Infinium HumanMethylation450 BeadChip (450k array); (2) TCGA Stomach Cancer (STAD), comprised of 395 samples using the Illumina Human Methylation 450k Array; (3) TCGA Stomach Cancer (STAD), comprised of 48 samples using the Illumina HumanMethylation27 array (27k array). The following Gene Expression Omnibus (GEO) methylation profile studies were collected: (4) GSE72872 comprised of 125 samples using the Illumina Human Methylation 450k Array (GPL13534); (5) GSE30601, comprised of 297 samples using the Illumina Human Methylation 27k Array; (6) GSE32925, comprised of 16 samples using the Illumina Human Methylation 27k Array; (7) GSE81334, comprised of 23 samples using the Illumina Human Methylation 450k Array (GPL13534); (8) GSE25869, comprised of 32 samples using the Illumina Human Methylation 27k Array; and (9) GSE31788, comprised of 54 samples using the Illumina Human Methylation 27k Array. In total, 366 samples were labelled as esophageal adenocarcinomas and 853 were labelled as stomach adenocarcinomas.

The discovery dataset was comprised of the two cohorts, TCGA ESCA 450k and TCGA STAD 450k and 27k. The validation dataset was comprised of the remaining cohorts, GSE72872, GSE30601, GSE32925, GSE81334, GSE25869, and GSE31788.

We retrieved the TCGA 450k datasets, (1) and (2), in their raw intensity data (IDAT) file format along with their associated clinical data records. The raw IDAT files were first processed and quality controlled using the *minfi* and *limma* bioconductor packages, and the workflow developed for methylation analysis by Maksimovic et al. [23]. They were then normalized to create M values and beta values of each probe. After normalization, each probe was mapped to University of California Santa Cruz (UCSC) Gene IDs (mapping provided by the minfi bioconductor package). Probes that were not part of the Illumina Human Methylation 27k array probes were deliberately removed, leaving 18,433 unique probes with their associated genes. With regards to the remaining cohorts, we retrieved them in their processed forms (beta values) along with their associated clinical records. To address batch effects, the values of the samples in all the cohorts were z-scored.

### 2.2. Multiple Survival Screening (MSS) Methodology and Optimization

**Figure 1:**
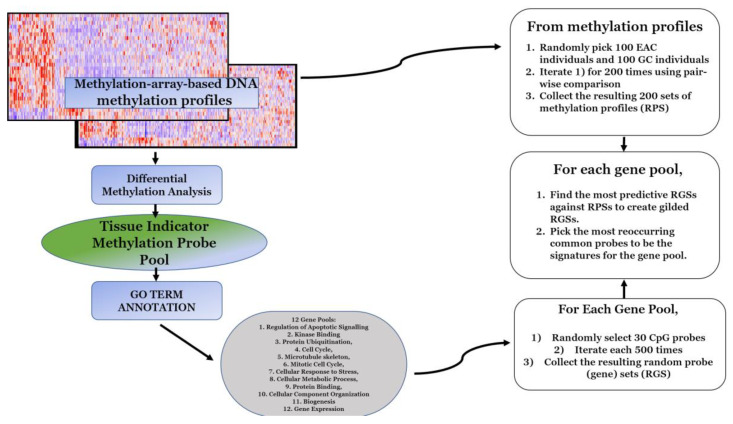
Diagram depicting the workflow used within the methodology. For greater detail in each step, refer to methods for dataset and further information.

Based on the study of Li et al. [6] and the optimizations described by Feng et al. [8], we used the following random sampling-focused methodology in our discovery dataset (Figure 1).

1. In the discovery dataset, probes that demonstrated significantly differential methylation profiles between the subgroup of esophageal carcinomas and the subgroup of stomach adenocarcinomas were selected to form a pool.
  - Significance was first defined by a probe-wise analysis performed using the limma Bioconductor package pipeline to calculate moderated t-statistics.
  - In original MSS methodology, statistical significance was determined by a *p*-value less than 0.05 and the application of other hyperparameters in downstream steps, while Feng et al. [8] suggested to optimize this step and choose the most significant 300–500 genes at this step. Thus, probes that had a false discovery rate (FDR)-corrected *p*-value of less than 0.0001 and a FC (fold change) greater than 3 were selected to form a gene pool of 536 unique probes.
2. Gene pools were annotated for GO (gene ontology) terms by the Database for Annotation, Visualization and Integrated Discovery (DAVID) [24]. For the given probe pool, we partitioned genes with replacement into GO-defined subpools. In the subgroup of cellular response to DNA damage stimulus comprised of 54 genes, similarly, the numbers of genes were as the following for other subgroups: Regulation of apoptotic signaling (38), kinase binding (35), protein ubiquitination (47), cell cycle (87), microtubule skeleton (59), mitotic cell cycle (58), cellular response to stress (95), cellular metabolic process (377), protein binding (376), cellular component organization or biogenesis (252), and gene expression (210).
3. Following the optimized MSS methodology proposed by Feng et al. [8], for a given GO-defined subpool, 500 random gene sets were created for each GO term. We then created 200 random patient sets (RPS) and to ensure an RPS was not dominated by a single subtype of stomach cancer we performed the following:
  - We obtained assigned subtypes for samples in the TCGA STAD cohort: Epstein–Barr virus (EBV), microsatellite instability (MSI), genomic stability, and chromosomal instability (CIN).
  - Twenty-five samples were randomly drawn from each subtype to obtain 100 samples of evenly distributed subtypes in each RPS.
  - Then, 100 samples of esophageal carcinoma were drawn and added to each RPS to create a balanced 200 sample RPS ratio of 1:1 stomach adenocarcinoma to esophageal carcinoma.
  - Then, 1,000,000 RPSs were created. A pairwise comparison was then performed to select the 200 most dissimilar RPSs. This was performed on a standard laptop in <8 hours.
4. Each random gene set (RGS) was then tested against all 200 RPSs: Fisher’s tests were used to determine if the RGS enriched the RPSs. This took <3 hours on a standard laptop. The *p*-values yielded by the Fisher’s tests were recorded and the reciprocal of their average was considered as the enrichment score of the RGS. For each GO term, the top 50 most significant RGSs were selected to be “gilded RGSs” based on the enrichment score. According to Feng et al. [8], the threshold for choosing the genes in the gilded RGSs could be chosen freely as it did not affect the downstream results.
5. The unique 30 most frequently appearing genes across the gilded RGSs of a GO term were then drawn as the set of signature genes for the corresponding GO term.

### 2.3. Gene Set Selection

Combinations of gene sets were tested using the discovery dataset, consisting of 185 esophageal cancer samples and 395 gastric cancer samples, and the validation dataset, comprising of 125 esophageal cancer samples and 370 gastric cancer samples. Prediction of labels for each sample was performed through the following processes:

1. The sample in question would be removed from the dataset.
2. For each GO term, we used the 30 signature genes to translate methylation profiles of patients in the training dataset into 1D vectors of shape (30, 1).
3. Centroids of each cluster of samples (esophageal or stomach) were then calculated based on the GO term vector.
4. The sample was then reintroduced to the dataset and assigned to a centroid based on the GO term vector.
5. This was performed for each sample in each dataset to evaluate the prediction and recall accuracy of each signature set.

### 2.4. Probe Distance to Transcription Start Site Calculation

The best performing signatures were then further investigated to calculate their probes’ chromosomal positions. The nearest downstream transcription start sites (TSS) of each probe was obtained from the Infinium methylation EPIC Array manifest. Chromosomal map coordinates of each TSS and probe were determined by the Encyclopedia of DNA Elements (ENCODE) consortium.

1. The midpoint of each transcription start site was calculated and represented the TSS location.
2. The distance from the midpoint of the TSS to the probe in question was calculated.
3. The average distance from TSS to probe for each signature was then calculated.

## 3. Results

### 3.1. Methylation Signatures for Differentiation between Gastric and Esophageal Adenocarcinomas

To identify an effective and generalizable methylation signature for predicting whether a tumor found in the gastroesophageal tract contains gastric carcinoma attributes or esophageal carcinoma attributes, we constructed a discovery dataset that was comprised of methylation array data by two cohorts, TCGA ESCA and TCGA STAD, referred to as T1, where in total 628 samples were acquired (nEC = 185, nGC = 443; EC, esophageal cancer; GC, gastric cancer). Similarly, an independent validation dataset was formed using the methylation array data from the cohorts of GSE72872, GSE30601, GSE32925, and GSE81334 (nEC = 164, nGC = 383; referred to as V1). The clinical characteristics of T1 and V1 are shown in Table 1 and Table 2. Both T1 and V1 were beta-normalized within each cohort and then z-score normalization was applied across all cohorts to address batch effects between the cohorts.

We performed a differential methylation analysis of the 5′-...Cytosine-Phosphate-Guanine...-3′ (CpG) probes between gastric cancer samples and esophageal cancer samples (Table 3), which revealed that over half of the significantly differentially methylated (Fold Change >3, *p*-value < 0.0001) probes were gene promoter-associated, making them ideal for filtering with 27k array (a promoter-based array). Our final pool of probes consisted of 18,433 differentially methylated CpG islands, each of which was associated with the promoter of a gene.

Implementing a methodology based on Multiple Survival Screening (MSS) [6] and using the optimizations demonstrated by Feng et al. [8], which is a computational random search scheme that can identify reliable signature genes, we obtained 12 methylation signatures from T1 corresponding to 12 groups of GO terms closely associated with critical cellular processes (Table 4). Each signature set contained 30 unique methylation probes and was used to translate a given methylation profile into a feature vector. We tested the 12 signatures against T1 and V1 and observed the predictive ability of each signature using the leave-one-out method. Precision and recall rates for each methylation signature set were determined, where a true positive prediction was defined as predicting a gastric tumor sample to be so, and a false positive prediction to be an esophageal cancer tumor (Table 5). Of the 12 methylation signatures, precision and recall rates for the protein binding, gene expression, and cellular component organization gene set, methylation signatures performed best against V1, achieving precision and recall rates of 94.7% and 99.2%, 97.6% and 96.8%, and 96.8% and 97.6%, respectively. These same signatures also performed best against T1 (Table 5).

**Figure 2:**
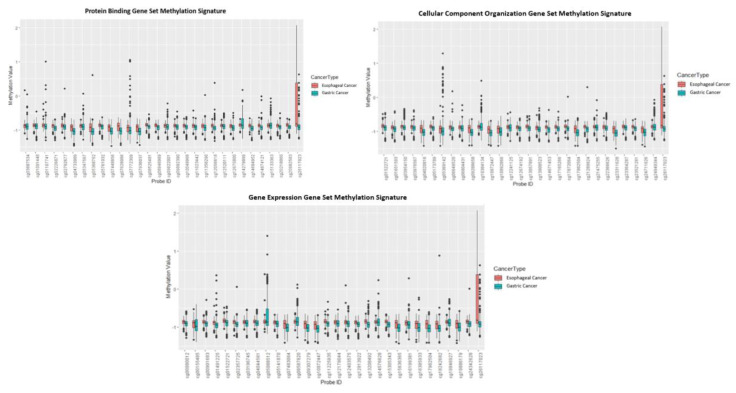
Box plots’ methylation values for all methylation probes in the best performing methylation signatures distinguishing esophageal cancer vs. gastric cancer.

The best performing signatures were further investigated, and their probes and associated genes are listed in Table 6. Each probe’s chromosomal position was used to calculate its distance to its downstream TSS (Tables S9–S11). The methylation sites of the signatures were very close (i.e., 170–270 base pairs) to their downstream TSSs. Evidently, all 30 of the probes in each methylation signature either increased or decreased in their methylation, depending on the tumor tissue, indicating that the genes they are associated with may consequently have their level of expression impacted, which may contribute to the differentiation of the two types of cancers. Additionally, a boxplot was generated to visualize differential methylation scores of the methylation probes of the three signatures between esophageal and gastric cancer types (Figure 2).

### 3.2. Utilization of Optimized Methodology to Use Fewer Computational Resources

The original MSS methodology essentially relied on random searching, which was implemented through randomly generating sets of methylation sites, ranking their ability to represent a tumor sample, and selecting consensus genes from top-ranked methylation site sets to serve as methylations’ signatures in the predictor. This process is incredibly computationally demanding and had undefined hyperparameters that accounted for the number of total iterations as well as ranking criteria.

This is the first study, to our knowledge, to utilize Feng et al.’s optimizations on the MSS [8]. We were able to perform the methylation signature discovery on a regular laptop within a reasonable time frame. Firstly, we substituted the hyperparameters that determine the base “gene pool” of random sampling by simply picking the 500 most significantly differentially methylated sites. We then introduced one single threshold (see Section 2. Methods). This dramatically reduced the 1 million iterations required by the original methodology to 20,000 iterations, while retaining the same prediction power. The implementations of the methodology optimized by Feng et al. [8] are suitable for a small computational resource such as a standard laptop with MSS capable of being performed within a day.

## 4. Discussion

The goal of precision medicine is to treat patients on an individual level, taking into consideration their individual lifestyle, characteristics, and genetics, rather than a “one-size-fits-all” approach. This has been demonstrated in other studies that have used genomic DNA variations to predict phenotypic characteristics of tumors [25]. In terms of GEJ cancer, incorporating precision oncology would mean moving towards greater molecular characterization of GEJ tumors, allowing for more informed decisions by clinicians regarding optimal treatment options for individuals. The development of a robust methylation signature for distinguishing cancer types that GEJ tumors could consist of (gastric and/or esophageal cancers) could, perhaps, improve accuracy of staging assignments of patients where these cancer types can differ based on the staging convention that is used. In this study, we reported three robust methylation signature sets that can accurately predict and characterize tumors found in the gastroesophageal tract as either of a gastric or esophageal cancer nature.

In this study, the optimized version of the Multiple Survival Screening (MSS) algorithm was used which was initially developed by our group [6,8]. The original MSS algorithm was developed to identify cancer prognostic markers with flagship sensitivity and specificity [6]. However, by amending and optimizing steps to the algorithm pipeline, an improvement to processing speeds was shown along with the production of robust sets of markers comparable to markers generated by the original algorithm [8]. The algorithm was employed to nine DNA methylation datasets which were curated and partitioned into both a training and a validation set to identify methylation signatures capable of characterizing GEJ tumors using methylation profiles. While 12 methylation signatures were identified, each with its own associated gene sets of critical cellular processes, most did not perform to practical use standards on the validation set. However, three of the signatures performed well on both the discovery and validation sets. Protein binding, gene expression, and cellular component organization gene set methylation signatures performed best against unseen data, achieving very high precision and recall rates, indicating their high accuracy and demonstrating their high predictive power. In terms of precision, the gene expression gene set methylation signature performed best against the validation data, achieving a precision rate of 97.6%. In terms of recall rate, protein binding gene set methylation signature performed best against the validation data, achieving a recall rate of 99.5%. With precision and recall rates aligning with their conventional definitions, the gene expression methylation signature is the best in terms of determining if a tumor in the gastroesophageal tract contained more gastric attributes and, thus, could be treated as so, which is what the methodology originally set out to do. However, the protein binding signature also performed well in terms of precision, with a rate of 94.7%, and was near flawless in its ability to correctly distinguish the tumor types.

For all three signatures, each probe corresponded to a CpG dyad located somewhere along the promoter region of a gene. The methylation sites of the signatures were very close (i.e., 170–270 bp) to their downstream transcription start sites (TSS), suggesting that the methylations near TSSs play much more important roles in tumorigenesis. With the methylation levels of these promoter-associated dyads changing between the different types of cancer, this may impact the associated gene’s expression levels. Thus, the role of the associated genes in their contribution to differentiating between different cancer types warrants investigation in future studies. This could prove to be useful in understanding the nature of gastroesophageal cancers as well as providing potential novel therapeutic targets.

The methylation signatures identified in this study showcased a novel approach to characterizing and predicting GEJ tumors through methylation profiles with a high predictive power, which have been largely understudied altogether. As methylation technology improves and becomes more popular, methylation signatures will become more widely used and, consequently, this signature could have an impact in a clinical setting. The shift from conventional tumor staging and diagnostic techniques towards more objective and precise molecular characterizations presents the need for sensitive and accurate molecular characterizations of gastroesophageal tumors for use in the clinical setting. Under the conventions of UICC-AJCC, all GEJ tumors would be characterized under esophageal tumors, hindering the efficacy and specificity of a treatment and therapy. Therefore, our signatures provide a large improvement to clinical misclassifications and corresponding treatments considering correct molecular characterizations of GEJ tumors through personalized oncology. Each of the identified signatures have a high predictive power and accuracy to classify a GEJ tumor as gastric or esophageal. Each signature includes 30 methylation probes associated with CpG sites on the promoter regions of genes associated with protein binding, cellular component organization, or gene expression, which were discovered using the less computationally taxing version of the Multiple Survival Screening algorithm, as described previously [6]. To our knowledge, this is the first study to provide a methylation signature to characterize gastroesophageal tumors in the gastroesophageal tract. Furthermore, this is the first study to provide precision and recall rates of a molecular signature to characterize gastroesophageal tumors. The benefit of a classification tool such as this is that a correct classification of the tumor can lead to a correct diagnosis, which will inform a more accurate prognosis and a more effective treatment plan.

The signature can be used in a clinical setting in a relatively quick manner to help inform clinicians of the predicted type of gastroesophageal tumor and, consequently, the best course of therapy to provide. A tumor sample will be obtained and have its DNA derived and then sequenced using a targeted DNA methylation approach [26]. A targeted methylation panel can be designed to interrogate only the probes corresponding to those in our three signatures to provide a more informative, rapid, and cost-effective test. It is necessary that a tumor sample contains a high fraction (i.e., >70%) of cancer cells as opposed to a mix with normal tissue to ensure the reliability of our signatures’ predictions. This is a similar requirement of many common commercial molecular sequencing-based oncology tests [27,28]. The methylation scores of the specific probes that belong to the signatures can be obtained and then compared to an existing database of esophageal cancer and gastric cancer tumor samples. The signature will predict whether a tumor is more likely of gastric or esophageal cancer origin, aiding the clinicians in a consequent determination of therapy.

The robustness of our signatures was demonstrated through their consistently strong precision and recall rates across a total of eight independent studies. Our differential methylation analysis of the probes revealed that over 50% of the significantly differentially methylated probes (*p* < 0.0001 and FC > 3) were promoter associated, justifying the filtering of the probes to contain only probes found in the 27k array. The benefit of having the probes in our signature in the 27k array is that the signature can be applied to the three most popular methylation arrays including the Illumina 450k, the Illumina 27k, and the EPIC 850k. While utilizing probes only found in the 27k array decreases the number of differentially methylated probes we were able to use, we aimed to create a very generalized signature that allows the use of any of the aforementioned methylation arrays when assessing a tumor without straining a clinician for resources, including the Illumina 27k methylation array, which is still in use. As the 450k array grows in popularity and the use of the 27k array becomes obsolete, an improved signature can be investigated with the use of all 450k probes as opposed to just the 27k. However, with a growing use of methylation arrays, the signature can be validated on future datasets to test its robustness.

## 5. Conclusions

In conclusion, we identified the first sets of methylation signatures with a high predictive power for the classification of gastroesophageal tumors to suggest the inclusion of an additional criterion to categorize these tumors for the improvement of diagnosis for individual patients. With the greater confidence in diagnosis of the cancer tumor type, more accurate prognosis and treatments will follow as a result.

## Figures and Tables

**Table 1:** The Cancer Genome Atlas (TCGA) training set clinical characteristics.

Training Set (N = 628)		
Sex	Male	443
	Female	185
	Unknown	0
Age (yrs.)	Range	27–90
	Mean	64.7
	Unknown	5
Stage	I	54
	II	136
	III	286
	IV	124
	Unknown	28

For additional information pertaining to each study refer to Tables S1 and S2.

**Table 2:** Validation set clinical characteristics composed of GSE72872, GSE30601, GSE32925, GSE81334, GSE25869, and GSE31788 datasets.

Validation Set (N = 548)		
Sex	Male	391
	Female	139
	Unknown	18
Age (yrs.)	Range	23–92
	Mean	64.5
	Unknown (No. of patients)	43

For additional information pertaining to each study refer to Tables S3–S8.

**Table 3:** Results for the differential expression of methylation probes across TCGA gastric cancer and esophageal cancer.

Differentially Methylated Probes (DMPs) (FC >3, *p*-value < 10^−4^)	N = 81814
Differentially Methylated Regions	N = 28054
DMPs overlapping with 27k Array	N = 536
Regulatory Feature Group	Percentage of DMPs
Promoter Associated	81.3%
Gene Associated	0.25%
Gene Associated Cell Specific	0.55%
Relation to Island	Percentage of DMPs
OpenSea	18.4%
Island	55.0%
N_Shore	11.5%
S_Shore	9.7%

FC, Fold Change

**Table 4:** Gene ontology (GO) analysis of differentially methylated probes to pool together for signature sets.

GO Term	Fold Enrichment	FDR	GO Accession Number
Single-multicellular organism process	1.0508656015	0.82 × 10^−7^	0044707
Anatomical structure morphogenesis	1.083136144	3.45 × 10^−6^	0009653
Single-organism developmental process	1.050520832	6.97 × 10^−6^	0044767
Anatomical structure development	1.050309311	8.2 × 10^−6^	0048856
Cell fate commitment	1.281284221	2.60 × 10^−5^	0045165
Epithelium development	1.1318538	2.65 × 10^−5^	0060429
Developmental process	1.047949197	1.32 × 10^−5^	0032502
Organ morphogenesis	1.13549378	3.45 × 10^−5^	0009887
Tissue development	1.097778353	5.93 × 10^−5^	0009888
Skeletal system development	1.187655599	1.30 × 10^−4^	0001501
Multicellular organism development	1.050805467	1.45 × 10^−4^	0007275
Tube development	1.157944133	0.001406	0035295

**Table 5:** Methylation signatures’ precision and recall.

Methylation Signature	Training Set (nEC = 185, nGC = 443)	Validation Set (nEC = 164, nGC = 383)
Protein Binding	Precision: 99.5% Recall: 96.6%	Precision: 94.7% Recall: 99.2%
Cellular Component Organization	Precision: 98.5% Recall: 98.0%	Precision: 96.8% Recall: 97.6%
Gene Expression	Precision: 98.0% Recall: 98.5%	Precision: 97.6% Recall: 96.8%

**Table 6:** Protein binding, cellular component organization, and gene expression methylation signatures’ probes, associated genes, and gene descriptions.

Cellular Component Biogenesis			Gene Expression			Protein Binding		
Probe	Gene	Gene Description	Probe	Gene	Gene Description	Probe	Gene	Gene Description
cg26117023	LOXL3	Lysyl Oxidase Like 3	cg00901683	CPSF4	Cleavage and Polyadenylation Specific Factor 4	cg08946989	TBC1D7	TBC1 Domain Family Member 7
cg04020816	MAN2A1	Mannosidase Alpha Class 2A Member 1	cg01491225	ZCCHC9	Zinc Finger CCHC-Type Containing 9	cg01091448	AMACR	Alpha-Methylacyl-CoA Racemase
cg21475255	DAG1	Dystroglycan 1	cg11225935	KDM5A	Lysine Demethylase 5A	cg09892390	ARHGAP21	Rho GTPase Activating Protein 21
cg23364287	IP6K2	Inositol Hexakisphosphate Kinase 2	cg14576628	PRMT1	Protein Arginine Methyltransferase 1	cg01107741	CANT1	Calcium Activated Nucleotidase 1
cg01651593	CDC20	Cell Division Cycle 20	cg00155485	MED13L	Mediator Complex Subunit 13L	cg03887534	BCL2L13	BCL2 Like 13
cg05173789	RPLP0	Ribosomal Protein Lateral Stalk Subunit P0	cg08587820	BHLHE40	Basic Helix-Loop-Helix Family Member E40	cg05368762	TMBIM6	Transmembrane BAX Inhibitor Motif Containing 6
cg09288658	ZAK	Mitogen-Activated Protein Kinase Kinase Kinase 20	cg12403575	TRADD	TNFRSF1A Associated Via Death Domain	cg26117023	LOXL3	Lysyl Oxidase Like 3
cg06649520	ARFIP1	ADP Ribosylation Factor Interacting Protein 1	cg12179044	GCN1L1	GCN1 Activator of EIF2AK4	cg05761032	CCPG1	Cell Cycle Progression 1
cg10384134	RPS9	Ribosomal Protein S9	cg12813922	RAB3GAP1	RAB3 GTPase Activating Protein Catalytic Subunit 1	cg07448856	ZNF670	Zinc Finger Protein 670
cg10872447	GTF2F2	General Transcription Factor IIF Subunit 2	cg17982504	DDX28	DEAD-Box Helicase 28	cg07628086	AP2B1	Adaptor Related Protein Complex 2 Subunit Beta 1
cg14671453	STX4	Syntaxin 4	cg19846927	MRPL44	Mitochondrial Ribosomal Protein L44	cg09822001	APOA1BP	NAD(P)HX Epimerase
cg17982504	DDX28	DEAD-Box Helicase 28	cg19886179	PSMD14	Proteasome 26S Subunit, Non-ATPase 14	cg10049968	FAM219A	Family with Sequence Similarity 219 Member A
cg21289924	EIF3A	Eukaryotic Translation Initiation Factor 3 Subunit A	cg02357725	IMP3	IMP U3 Small Nucleolar Ribonucleoprotein 3	cg11356290	AZI2	5-Azacytidine Induced 2
cg01522721	MIR1181	MicroRNA 1181	cg05141870	MIR423	MicroRNA 423	cg12520111	PPIA	Peptidylprolyl Isomerase A
cg03954150	C18orf55	Translocase of Inner Mitochondrial Membrane 21	cg07483064	ENO1	Enolase 1	cg12675800	TRAPPC6B	Trafficking Protein Particle Complex 6B
cg03976567	AKD1	Adenylate Kinase 9	cg09307279	GLT8D1	Glycosyltransferase 8 Domain Containing 1	cg14874121	HSD17B4	Hydroxysteroid 17-Beta Dehydrogenase 4
cg05369142	ALS2CL	ALS2 C-Terminal Like	cg10872447	GTF2F2	General Transcription Factor IIF Subunit 2	cg20218060	CLK1	CDC Like Kinase 1
cg06804431	GNRHR2	Gonadotropin Releasing Hormone Receptor 2 (Pseudogene)	cg13208492	TSN	Translin	cg20982583	POLR2F	RNA Polymerase II Subunit F
cg10892866	PYGO2	Pygopus Family PHD Finger 2	cg15305343	NSUN4	NOP2/Sun RNA Methyltransferase 4	cg02226871	VPS28	Vacuolar Protein Sorting-Associated Protein 28 Homolog
cg12241125	EIF4H	Eukaryotic Translation Initiation Factor 4H	cg15636365	PNPLA7	Patatin Like Phospholipase Domain Containing 7	cg02792677	MRPL4	Mitochondrial Ribosomal Protein L4
cg12674192	MAK16	MAK16 Homolog	cg16199381	TSTD2	Thiosulfate Sulfurtransferase Like Domain Containing 2	cg04733989	NAGA	Alpha-N-Acetylgalactosaminidase
cg13057891	ERCC5	ERCC Excision Repair 5, Endonuclease	cg16385933	PDCD4	Programmed Cell Death 4	cg05347567	ZC3H10	Zinc Finger CCCH-Type Containing 10
cg13908523	PRKCD	Protein Kinase C Delta	cg18242682	FOXK2	Forkhead Box K2	cg07772309	NELF	NMDA Receptor Synaptonuclear Signaling and Neuronal Migration Factor
cg17165266	KRT18	Keratin 18	cg24342628	KDM1B	Lysine Demethylase 1B	cg07936037	SSR1	Signal Sequence Receptor Subunit 1
cg17872064	NOP58	NOP58 Ribonucleoprotein	cg26117023	LOXL3	Lysyl Oxidase Like 3	cg08525481	OGFR	Opioid Growth Factor Receptor
cg22366626	ZFYVE20	Rabenosyn, RAB Effector	cg00080012	EED	Embryonic Ectoderm Development	cg11023442	PITPNA-AS1	PITPNA antisense RNA 1
cg23311628	RAB8B	RAB8B, Member RAS Oncogene Family	cg01522721	CDC37	Cell Division Cycle 37	cg12056618	KLF13	Kruppel Like Factor 13
cg23521281	WDR75	WD Repeat Domain 75	cg03196745	ISCU	Iron-Sulfur Cluster Assembly Enzyme	cg14279899	IFNGR1	Interferon Gamma Receptor 1
cg24711626	KIAA1012	Trafficking Protein Particle Complex 8	cg04044561	POP7	POP7 Homolog, Ribonuclease P/MRP Subunit	cg14694952	HTT	Huntingtin
cg24949344	ANO6	Anoctamin 6	cg05088512	DKKL1	Dickkopf Like Acrosomal Protein 1	cg15133363	HILPDA	Hypoxia Inducible Lipid Droplet Associated

## Supplementary Materials

The following are available online at https://www.mdpi.com/2072-6694/12/5/1208/s1, Table S1: Clinical characteristics of the samples in TCGA STAD (stomach adenocarcinoma), Table S2: Clinical characteristics of the samples in TCGA-ESCA (esophageal adenocarcinoma), Table S3: Clinical characteristics of the samples in GSE81334, Table S4: Clinical characteristics of the samples in GSE32925, Table S5: Clinical characteristics of the samples in GSE30601, Table S6: Clinical characteristics of the samples in GSE72872, Table S7: Clinical characteristics of the samples in GSE31788, Table S8: Clinical characteristics of the samples in GSE25869, Table S9: Positions of the gene methylation signature (i.e., gene expression) probes on chromosomes, Table S10: Positions of the gene methylation signature (i.e., protein-binding signature) probes on chromosomes, Table S11: Positions of the methylation signature (cellular component organization signature) probes on chromosomes.
