# Supplementary material for: Methylation-Based Signatures for Gastroesophageal Tumor Classification
Source: Cancers (Basel). 2020 May 11;12(5):1208. doi: 10.3390/cancers12051208 (PMC7281220; doi:10.3390/cancers12051208)
Supplement: Supplementary file 1 [file cancers-12-01208-s001.pdf]

# Supplementary Material: Methylation-Based Signatures for Gastroesophageal Tumor Classification

**Nikolay Alabi, Dropen Sheka, Ashar Siddiqui and Edwin Wang**

**Table S1.** Clinical characteristics of the samples in TCGA STAD (Stomach Adenocarcinoma).

| Gastric Cancer (N = 443) | | |
|---|---|---|
| Sex | Male | 285 |
| | Female | 158 |
| Age (yrs.) | Range | 30–90 |
| | Mean | 65.7 |
| | Unknown | 5 |
| Stage | I | 33 |
| | II | 93 |
| | III | 198 |
| | IV | 119 |

**Table S2.** Clinical characteristics of the samples in TCGA-ESCA (Esophageal Adenocarcinoma).

| Esophageal Cancer (N = 185) | | |
|---|---|---|
| Sex | Male | 158 |
| | Female | 27 |
| Age (yrs.) | Range | 27–90 |
| | Mean | 62.5 |
| | SD | 11.9 |
| Stage | I | 31 |
| | II | 43 |
| | III | 88 |
| | IV | 4 |
| | Unknown | 17 |

Missing clinical characteristics: 17

**Table S3.** Clinical characteristics of the samples in GSE81334.

| GSE81334 EAC (N=30) | | |
|---|---|---|
| Sex | Male | 26 |
| | Female | 4 |
| Age (yrs.) | Mean | 65.6 |
| | SD | 13.3 |
| | Range | 35–88 |

**Table S4.** Clinical characteristics of the samples in GSE32925.

| GSE32925 (EAC) (N = 24) | | | |
|---|---|---|---|
| EAC | Sex | Male | 20 |
| | | Female | 4 |
| | Age (yrs.) | Mean | 71 |
| | | Median | 75 |
| | | Range | 44–87 |
| | Differentiation | Well | 1 |
| | | Moderate | 9 |
| | | Poor | 13 |
| | | Unknown | 1 |
| | Neoadjuvant chemotherapy | Yes | 8 |
| | | No | 15 |
| | | Unknown | 1 |

**Table S5.** Clinical characteristics of the samples in GSE30601.

| GSE30601 (STAD) (N = 188) | | |
|---|---|---|
| Sex | Male | 121 |
| | Female | 67 |
| Age (yrs.) | Mean | 63 |
| | Unknown (No. of Patients) | 23 |
| | Range | 23–92 |
| Lauren Classification | Intestinal | 97 |
| | Diffuse | 71 |
| | Mixed | 20 |
| Differentiation | Undifferentiated | 2 |
| | Poorly | 114 |
| | Moderately | 67 |
| | Well | 5 |
| Stage | I | 28 |
| | II | 29 |
| | III | 70 |
| | IV | 61 |

**Table S6.** Clinical characteristics of the samples in GSE72872.

| GSE72872 (EAC) (N = 125) | | |
|---|---|---|
| Sex | Male | 111 |
| | Female | 9 |
| | Unknown | 5 |
| Age (yrs.) | Mean | 63 |
| | Unknown (No. of Patients) | 7 |
| | Range | 27–83 |
| Survival Months | Unknown | 8 |
| | Mean | 22 |
| | Range | 0–166 |
| Overall Survival | Deceased | 70 |
| | Alive | 47 |
| | Unknown | 8 |

**Table S7.** Clinical characteristics of the samples in GSE31788.

| GSE31788 STAD (N = 51) | | |
|---|---|---|
| Sex | Male | 38 |
| | Female | 13 |
| Age (yrs.) | Mean | 69.7 |
| | SD | 10.5 |
| Tumor Location in Stomach | Upper | 26 |
| | Middle | 12 |
| | Lower | 13 |
| Histology | Undifferentiated | 2 |
| | Intestinal Type | 24 |
| | Diffuse Type | 27 |
| Epigenotype | Low Epigenotype | 7 |
| | High Epigenotype | 13 |
| | $EBV^+$ Epigenotype | 11 |
| | Outlier | 20 |

**Table S8.** Clinical characteristics of the samples in GSE25869.

| GSE25869 STAD (N = 130) | | |
|---|---|---|
| Sex | Male | 75 |
| | Female | 42 |
| | Not Specified | 13 |
| Age (yrs.) | Age ≥60 | 65 |
| | Age < 60 | 52 |
| | Not Specified | 13 |
| Lauren Classification | Intestinal | 42 |
| | Diffuse | 80 |
| | Mixed | 8 |
| Stage | I | 30 |
| | II | 40 |
| | III | 42 |
| | IV | 18 |

**Table 9.** Positions of the gene methylation signature (i.e., gene expression) probes on chromosomes.

| Gene Expression Methylation Signature | | | | | | | |
|---|---|---|---|---|---|---|---|
| **Probes** | **Chromosome** | **Chromosomal Position** | **Strand Polarity** | **CpG Island Position** | **TSS Region** | **Midpoint of TSS** | Distance from the Probe to the TSS (bp) |
| cg00080012 | chr11 | 85955689 | + | chr11:85955808-85956517 | 11:85955412-85955756 | 85955584 | 105 |
| cg00155485 | chr12 | 116714600 | − | N/A | 12:116713773-116714905 | 116714339 | 261 |
| cg00901683 | chr7 | 99036756 | − | chr7:99036099-99036892 | 7:99035978-99037253 | 99036616 | 140 |
| cg01491225 | chr5 | 80597551 | − | chr5:80597320-80597734 | 5:80597082-80597998 | 80597540 | 11 |
| cg01522721 | chr19 | 10514734 | − | chr19:10513990-10515616 | 19:10514184-10515232 | 10514708 | 26 |
| cg02357725 | chr15 | 75932598 | + | chr15:75931952-75932782 | 15:75931730-75933616 | 75932673 | 75 |
| cg03196745 | chr12 | 108956112 | + | chr12:108956188-108956759 | N/A | N/A | N/A |
| cg04044561 | chr7 | 100303839 | − | chr7:100303014-100304079 | 7:100302794-100304160 | 100303477 | 362 |
| cg05088512 | chr19 | 49866817 | − | chr19:49866752-49867209 | 19:49865953-49867055 | 49866504 | 313 |
| cg05141870 | chr17 | 28444127 | + | chr17:28443746-28444011 | 17:28443425-28444742 | 28444084 | 43 |
| cg07483064 | chr1 | 8938751 | + | chr1:8938098-8939409 | 1:8938599-8939675 | 8939137 | 386 |
| cg08587820 | chr3 | 5020949 | + | chr3:5019876-5022223 | 3:5020258-5021202 | 5020730 | 219 |
| cg09307279 | chr3 | 52739927 | − | chr3:52739537-52740426 | 3:52739765-52740250 | 52740008 | 81 |
| cg10872447 | chr13 | 45694593 | − | chr13:45694522-45694966 | 13:45693645-45695587 | 45694616 | 23 |
| cg11225935 | chr12 | 498478 | − | chr12:498098-498920 | 12:497660-499377 | 498519 | 41 |
| cg12179044 | chr12 | 120632364 | + | chr12:120632222-120632784 | 12:120631688-120633300 | 120632494 | 130 |
| cg12403575 | chr16 | 67194943 | + | chr16:67195600-67195827 | 16:67193608-67195198 | 67194403 | 540 |
| cg12813922 | chr2 | 135809940 | − | N/A | 2:135809334-135810656 | 135809995 | 55 |
| cg13208492 | chr2 | 122513376 | − | chr2:122512987-122513526 | 2:122512427-122513814 | 122513121 | 255 |
| cg14576628 | chr19 | 50180360 | − | chr19:50180010-50180907 | 19:50179402-50180733 | 50180068 | 292 |
| cg15305343 | chr1 | 46806357 | − | chr1:46806339-46806836 | 1:46806004-46807143 | 46806574 | 217 |
| cg15636365 | chr9 | 140446240 | + | chr9:140445653-140446891 | 9:140445441-140447796 | 140446619 | 379 |
| cg16199381 | chr9 | 100396477 | − | chr9:100395340-100396414 | 9:100395246-100396751 | 100395999 | 478 |
| cg16385933 | chr10 | 112631792 | − | chr10:112631414-112632476 | 10:112631217-112633041 | 112632129 | 337 |
| cg17982504 | chr16 | 68056849 | + | chr16:68056582-68057353 | 16:68056456-68057707 | 68057082 | 233 |
| cg18242682 | chr17 | 80477962 | + | chr17:80477098-80478553 | N/A | N/A | N/A |
| cg19846927 | chr2 | 224821935 | + | chr2:224821928-224822575 | 2:224821664-224822528 | 224822096 | 161 |
| cg19886179 | chr2 | 162164993 | − | chr2:162164675-162165248 | 2:162164347-162165601 | 162164974 | 19 |
| cg24342628 | chr6 | 18155101 | − | chr6:18155534-18156354 | 6:18154981-18156722 | 18155852 | 751 |
| cg26117023 | chr2 | 74782096 | − | chr2:74781494-74782685 | N/A | N/A | N/A |

Mean distance from a probe to the TSS: 219.74bp

**Table S10.** Positions of the gene methylation signature (i.e., protein binding signature) probes on chromosomes.

| Protein Binding Methylation Signature | | | | | | | |
|---|---|---|---|---|---|---|---|
| **Probes** | **Chromosome** | **Chromosomal Position** | **Strand Polarity** | **CpG Island Position** | **TSS Region** | **Midpoint of TSS** | Distance from the Probe to the TSS (bp) |
| cg01091448 | chr5 | 34008127 | + | chr5:34007796-34008105 | 5:34007929-34008383 | 34008156 | 29 |
| cg01107741 | chr17 | 77006080 | − | chr17:77005746-77006106 | 17:77004720-77006501 | 77005611 | 469 |
| cg02226871 | chr8 | 145653662 | + | chr8:145653335-145654145 | 8:145652706-145654273 | 145653490 | 172 |
| cg02792677 | chr19 | 10362626 | − | chr19:10362771-10363384 | 19:10362293-10363456 | 10362875 | 249 |
| cg03887534 | chr22 | 18121194 | + | chr22:18121280-18122009 | 22:18120836-18122023 | 18121430 | 236 |
| cg04733989 | chr22 | 42467013 | − | chr22:42466407-42467121 | 22:42465847-42467456 | 42466652 | 361 |
| cg05347567 | chr12 | 56512408 | + | chr12:56511861-56512409 | 12:56511592-56512541 | 56512067 | 341 |
| cg05368762 | chr12 | 50135785 | + | chr12:50135029-50136043 | 12:50134864-50136182 | 50135523 | 262 |
| cg05761032 | chr15 | 55700743 | + | chr15:55700140-55700806 | 15:55699487-55701302 | 55700395 | 348 |
| cg07448856 | chr1 | 247242043 | + | chr1:247241579-247242201 | 1:247241168-247242553 | 247241861 | 182 |
| cg07628086 | chr17 | 33914258 | + | N/A | 17:33913804-33915203 | 33914504 | 246 |
| cg07772309 | chr9 | 140353475 | + | chr9:140352818-140354044 | N/A | N/A | N/A |
| cg07936037 | chr6 | 7313172 | + | chr6:7312811-7313656 | 6:7312455-7313987 | 7313221 | 49 |
| cg08525481 | chr20 | 61436809 | − | chr20:61435828-61437150 | N/A | N/A | N/A |
| cg08946989 | chr6 | 13328449 | + | chr6:13328587-13329096 | 6:13327892-13329387 | 13328640 | 191 |
| cg09822001 | chr1 | 156561334 | − | chr1:156561485-156561880 | 1:156560779-156561998 | 156561389 | 55 |
| cg09892390 | chr10 | 25013471 | − | chr10:25011963-25013816 | 10:25013339-25014110 | 25013725 | 254 |
| cg10049968 | chr9 | 34458533 | + | chr9:34457375-34458892 | 9:34458404-34458980 | 34458692 | 159 |
| cg11023442 | chr17 | 1420488 | − | chr17:1419442-1420434 | 17:1418501-1420594 | 1419548 | 940 |
| cg11356290 | chr3 | 28390998 | + | chr3:28389975-28390855 | 3:28389449-28391197 | 28390323 | 675 |
| cg12056618 | chr15 | 31619949 | + | chr15:31617763-31620908 | N/A | N/A | N/A |
| cg12520111 | chr7 | 44835727 | − | chr7:44835726-44836798 | 7:44835482-44836426 | 44835954 | 227 |
| cg26117023 | chr2 | 74782096 | − | chr2:74781494-74782685 | N/A | N/A | N/A |
| cg12675800 | chr14 | 39639447 | + | chr14:39639364-39639635 | 14:39638828-39640305 | 39639567 | 120 |
| cg14279899 | chr6 | 137540774 | + | chr6:137540126-137540678 | 6:137539473-137541132 | 137540303 | 471 |
| cg14694952 | chr4 | 3076907 | + | chr4:3075483-3075773 | N/A | N/A | N/A |
| cg14874121 | chr5 | 118788291 | − | chr5:118788125-118788428 | 5:118787812-118788968 | 118788390 | 99 |
| cg15133363 | chr7 | 128096265 | + | chr7:128095678-128096197 | 7:128095368-128096412 | 128095890 | 375 |
| cg20218060 | chr2 | 201729279 | + | chr2:201728892-201729577 | 2:201728531-201729972 | 201729252 | 27 |
| cg20982583 | chr22 | 38349717 | − | chr22:38349502-38349969 | 22:38348981-38350285 | 38349633 | 84 |

Mean distance from a probe to the TSS: 264.84bp

**Table S11.** Positions of the gene methylation signature (i.e., cellular component organization signature) probes on chromosomes.

| **Cellular Component Organization Methylation Signature** | | | | | | | |
|---|---|---|---|---|---|---|---|
| **Probes** | **Chromosome** | **Chromosomal Position** | **Strand Polarity** | **CpG Island Position** | **TSS Region** | **Midpoint of TSS** | Distance from the Probe to the TSS (bp) |
| cg01522721 | chr19 | 10514734 | − | chr19:10513990-10515616 | 19:10514184-10515232 | 10514708 | 26 |
| cg10872447 | chr13 | 45694593 | − | chr13:45694522-45694966 | 13:45693645-45695587 | 45694616 | 23 |
| cg17982504 | chr16 | 68056849 | + | chr16:68056582-68057353 | 16:68056456-68057707 | 68057082 | 233 |
| cg01651593 | chr1 | 43824241 | + | chr1:43824134-43825059 | 1:43823626-43825226 | 43824426 | 185 |
| cg03954150 | chr18 | 71815372 | − | chr18:71814555-71815716 | 18:71814856-71816018 | 71815437 | 65 |
| cg03976567 | chr6 | 110012095 | − | chr6:110012365-110012769 | 6:110011446-110013236 | 110012341 | 246 |
| cg04020816 | chr5 | 109025456 | − | chr5:109024965-109026701 | 5:109025415-109025826 | 109025621 | 165 |
| cg05173789 | chr12 | 120639210 | + | chr12:120638588-120639116 | 12:120638471-120639397 | 120638934 | 276 |
| cg05369142 | chr3 | 46735009 | − | chr3:46734731-46735717 | 3:46734119-46735928 | 46735024 | 15 |
| cg26117023 | chr2 | 74782096 | − | chr2:74781494-74782685 | N/A | N/A | N/A |
| cg06649520 | chr4 | 153700671 | − | chr4:153700539-153701491 | 4:153700103-153701806 | 153700955 | 284 |
| cg06804431 | chr1 | 145516254 | − | chr1:145516038-145516637 | 1:145515626-145517107 | 145516367 | 113 |
| cg09288658 | chr2 | 173940639 | − | chr2:173940414-173941271 | 2:173940347-173940855 | 173940601 | 38 |
| cg10384134 | chr19 | 54704842 | − | chr19:54704584-54705204 | 19:54704363-54705337 | 54704850 | 8 |
| cg10892866 | chr1 | 154934115 | − | chr1:154933928-154934735 | 1:154933351-154934958 | 154934155 | 40 |
| cg12241125 | chr7 | 73589459 | − | chr7:73588252-73589341 | N/A | N/A | N/A |
| cg12674192 | chr8 | 33342777 | − | chr8:33342171-33343141 | 8:33341954-33343211 | 33342583 | 194 |
| cg13057891 | chr13 | 103498411 | − | chr13:103498100-103498763 | 13:103497746-103499494 | 103498620 | 209 |
| cg13908523 | chr3 | 53194728 | + | chr3:53194716-53196133 | N/A | N/A | N/A |
| cg14671453 | chr16 | 31044716 | − | chr16:31044353-31045495 | 16:31044374-31045198 | 31044786 | 70 |
| cg17165266 | chr12 | 53342635 | + | chr12:53342805-53343162 | 12:53342459-53343010 | 53342735 | 100 |
| cg17872064 | chr2 | 203130438 | − | chr2:203130313-203130760 | 2:203130183-203131013 | 203130598 | 160 |
| cg21289924 | chr10 | 120839943 | + | chr10:120839592-120840480 | 10:120839482-120841154 | 120840318 | 375 |
| cg21475255 | chr3 | 49507186 | − | chr3:49507384-49508290 | 3:49507098-49508254 | 49507676 | 490 |
| cg22366626 | chr3 | 15140889 | + | chr3:15140035-15140890 | 3:15139617-15141482 | 15140550 | 339 |
| cg23311628 | chr15 | 63481868 | − | chr15:63481449-63481975 | 15:63481178-63482123 | 63481651 | 217 |
| cg23364287 | chr3 | 48754776 | − | chr3:48754577-48754904 | 3:48754397-48755486 | 48754942 | 166 |
| cg23521281 | chr2 | 190306491 | + | chr2:190306157-190306433 | 2:190305515-190306593 | 190306054 | 437 |
| cg24711626 | chr18 | 29522957 | + | chr18:29522401-29523577 | 18:29522116-29523924 | 29523020 | 63 |
| cg24949344 | chr12 | 45609653 | + | chr12:45609713-45610752 | 12:45609634-45609909 | 45609772 | 119 |

Mean distance from a probe to the TSS: 172.44 bp
